# Prospecting of exosomal-miRNA signatures as prognostic marker for gestational diabetes mellitus and other adverse pregnancy outcomes

**DOI:** 10.3389/fendo.2023.1097337

**Published:** 2023-02-09

**Authors:** Tridip Mitra, Richa Gulati, Anmol Uppal, Sajeetha R. Kumari, Saswati Tripathy, Priya Ranjan, Rajiv Janardhanan

**Affiliations:** ^1^ Department of Medical Research, Faculty of Medicine and Health Sciences, SRM Institute of Science and Technology, Kattankulathur, Tamil Nadu, India; ^2^ Navatar Group, Noida, Uttar Pradesh, India; ^3^ Department of Obstetrics and Gynaecology, SRM Institute of Science and Technology, Kattankulathur, Tamil Nadu, India; ^4^ Department of Electrical Engineering, Biju Patnaik University of Technology, Rourkela, Odisha, India

**Keywords:** clinical prognosis, exosomal microRNA, gestational diabetes mellitus, pregnancy outcomes, signaling pathways

## Abstract

Exosomal microRNA (ExomiRs) serves as potential cargo molecules responsible for post-translation of gene expression and intracellular communication playing a vital role in acting as clinically relevant prognostic biomarkers for identifying pregnancy-associated complications in patients. ExomiRs are associated with Gestational Diabetes Mellitus (GDM) as potential targets for understanding the pathophysiology of beta-cell dysfunction. ExomiRs (ExomiR 122, ExomiR 16-5p, ExomiR 215-5p, ExomiR 450b-3p, ExomiR 122-5p) aid to act as biomarkers and regulate the progression of diabetes and its related complication. These ExomiRshave been reported to interfere with the regulation of various genes such as ZEB2, IRS1, IRS2, GLUT1, GLUT4, etc. and inhibition of several pathways like PI3K/AKT, Wnt, and mTOR signaling pathways leading to the modulation in the development of GDM affecting the clinical and pathological features of women. These ExomiRs have also been associated with other pregnancy-associated complications, including preeclampsia, hypothyroidism, pregnancy loss, and ectopic pregnancies. On the other hand, overexpression of certain ExomiRs such as Exomir-515-5p, ExomiR-221, and ExomiR-96 serve a regulatory role in overcoming insulin resistance. Taken together, the current review focuses on the prospective capabilities of ExomiRs for diagnosis and clinical prognosis of GDM women with respect to pregnancy outcomes.

## Graphical Abstract

Depicts the graphical abstract of the article demonstrating the role of ExomiRs as potential biomarkers for the prognosis of GDM and related pregnancy outcomes as well as how AI-enabled algorithms can help in clinical resource allocation at the community level. Additionally, the arrow pointing down from AI-enabled niche-specific dashboards towards the automated clinical resource allocation is missing.

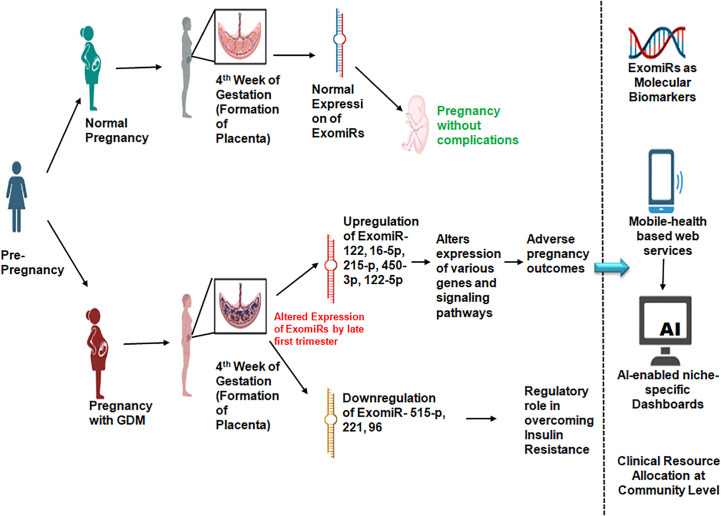

## Introduction

Exosomes are membranous vesicles produced in the endosomal compartment of most eukaryotic cells as a result of the lysosomal pathway and are usually 40-100 nm in size. These were discovered in 1983 (1) and were proposed to have no effect on neighboring cells and were considered either a cellular waste formed as a result of cell damage or a byproduct of cellular homeostasis until recently when they were found to act as complex cargo for delivering several proteins (2), lipids (3) and nucleic acids (2, 4) to the target cells (5), thereby playing a significant role in intercellular communication for serving pleiotropic cellular processes like signal transduction (6), immune responses (7) and antigen presentation (8). Thus, exosomes act as surrogate markers for different RNAs including microRNAs. These exosomes are shown to be released into the maternal circulatory system by the beginning of 6 weeks of pregnancy, i.e., the first trimester, and their concentration rapidly decrease within 48 hours postpartum  (9, 10) thereby acting as an early predictor of Gestational Diabetes Mellitus (GDM) (11). GDM is a transient diabetic condition that women develop during their pregnancy tenure occurring mainly due to hormonal changes and metabolic exigencies of pregnancy accompanied by genetic and environmental factors.

Exosomal microRNA (ExomiR) are 21-25 nucleotide long (12) nano-sized, non-coding RNA molecules serving pivotal regulatory roles in the progression of various diseases including insulin resistance in pregnant women. These ExomiRs can act as biomedical tools for a better prognosis of GDM and other pregnancy-associated complications like preeclampsia, preterm births, neonatal sepsis, etc (13). These molecules not only regulate but also act as significant biomarkers for several diseases, thereby helping to better diagnose the diseases. GDM can be related to preterm birth cases of Assisted Reproductive Technology (ART) (14), which is generally used to overcome infertility problems.

## Exosome biogenesis

Exosomes are vesicles formed by the process of endocytosis formed by the inward sprouting of the early endosome’s limiting membrane from Multivesicular Bodies (MVBs). The invagination of the inner membrane within MVBs results in the formation of Intraluminal Vesicles (ILVs). Nucleic material, transmembrane, and peripheral proteins are integrated into ILVs during their formation and accumulate in the MVB lumen, which later has two distinct fates: diffusion with lysosomes for degradation, or diffusion with the cytoplasmic membrane, which releases the vesicles to the extracellular space *via* exocytosis as exosomes (5, 15).

Exosome biogenesis and secretion are thought to be aided by either the ceramide-dependent pathway or the Endosomal Sorting Complex Required for Transport (ESCRT)-dependent pathway. ESCRT, which recognizes ubiquitin-related proteins, is the most well-known pathway. These pathways may involve sphingomyelinases, which are composed of four protein complexes, including ESCRT-0, -I, -II, and -III and the associated ATPase Vps4 complex. Proteins like ubiquitinylated proteins and clathrin are recruited for internalization by the ESCRT-0 complex subunits. ESCRT-I and II initiate the sprouting process and facilitated e-ubiquitination of cargo proteins carried out by enzymes, before the ILVs are formed, which are grouped to create larger membranous vesicles, MVBs, in the intracellular compartment. The last stage of membrane budding and partition is driven by the ESCRT-III complex (16).

The ceramide-dependent pathway serves as an alternative pathway for exosome formation. The ceramide-dependent pathway relies on the growth of glycolipoprotein micro domains (lipid rafts), where sphingomyelinases convert sphingomyelin into ceramide. The subsequent ceramide buildup causes micro domain fusion and starts the development of ILVs within MVBs (17, 18).

### Packaging of miRNA into exosomes

The miRNAs are an important requirement for exosomal cell signaling. During the biogenesis of exosomes, there are miRNAs present in the cell which are passed into the exosomes *via* a loading process, which is yet to be identified. It has been demonstrated that argonaute proteins play a crucial role in exosomal loading, miRNA transport, and miRNA function. RISC may not even be present at all in exosomal miRNAs. Instead, they are recognized by particular proteins, such as hnRNPA2B1 and hnRNPA1, which recognize the particular miRNA-binding motifs. As a result, the miRNAs are then loaded into exosomes in a selective manner (19).

**Figure 1:**
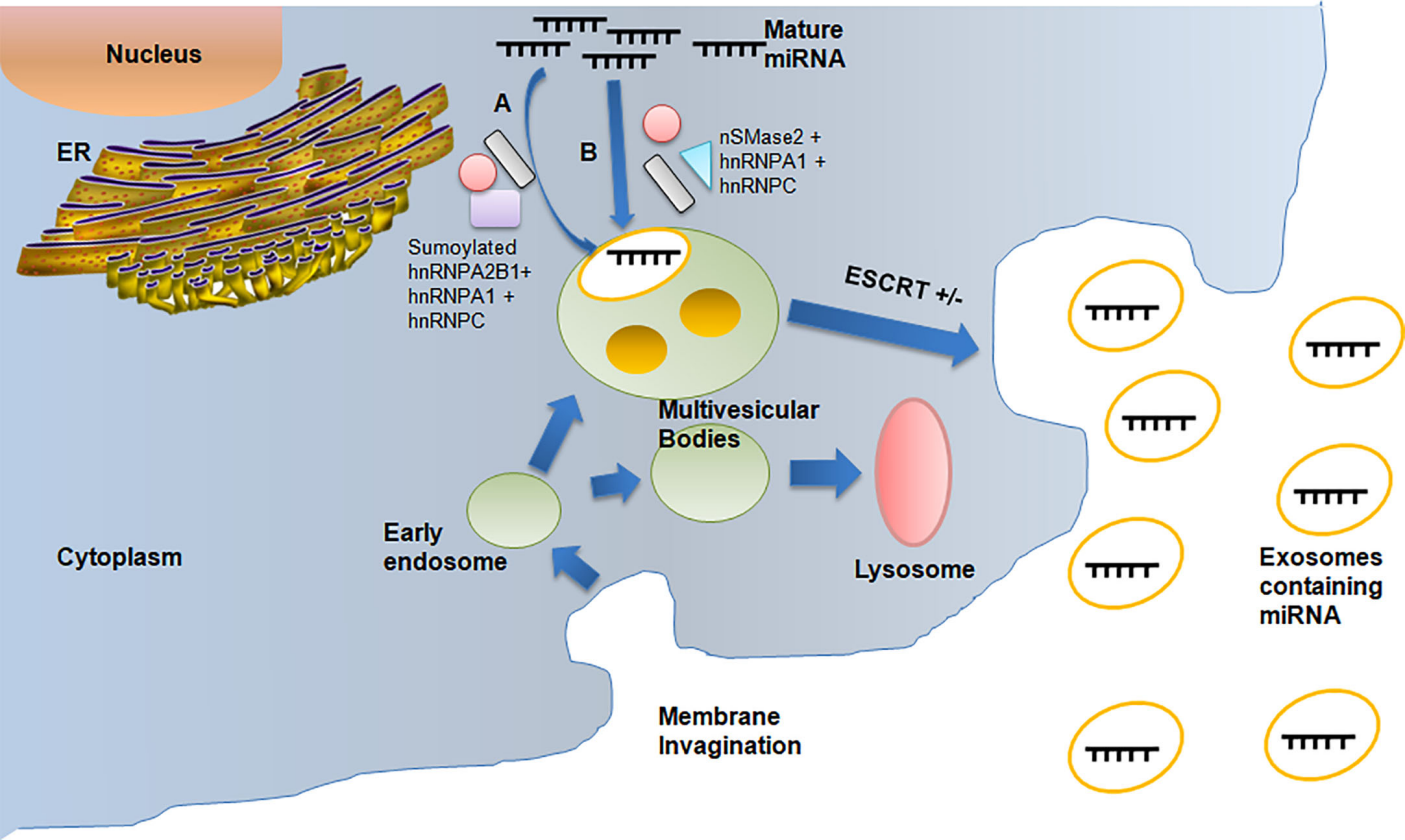
Depicts the packaging of miRNAs into exosomes during exosomal biogenesis resulting in the formation of ExomiRs, which helps in the biodelivery of miRNAs. **(A)** The miRNAs packaging during Exosome biogenesis occurs by sumoylated hnRNPA2B with the help of hnRNPA1 and hnRNPC. **(B)** Packagingof miRNA can also be done alternatively by nsMase2 with the help of hnRNPA1 and hnRNPC.

Although the underlying mechanisms of miRNA packaging are not fully understood, there are at least three putative methods for miRNA sorting into exosomes (**Figure 1**). Firstly, the pathway identified by Villarroya-Beltri et al.  (19) helps in the packing of selective miRNAs into exosomes by using sumoylated heterogeneous nuclear ribonucleoprotein A2B1 (hnRNPA2B1), which can detect the GGAG pattern found in the 3′-end portion of the miRNA sequences. Additionally, other two members of hnRNP family, hnRNPA1 and hnRNPC, which may bind to exomiRs and are thus implicated in the sorting process, may be involved in miRNA sorting. The second approach is by Kosaka et al. (20), in which the overexpression of neural sphingomyelinase 2 (nSMase2) is involved resulting in an increase in exosomal miRNA levels. While inhibition of nSMase2 expression resulted in fewer exosomal miRNAs, the hnRNPA1 and hnRNPC protein families, which bind to exosomal miRNAs, might be factors responsible for facilitating miRNA sorting. The third and final approach by Koppers-Lalicet al.  (21) deals with exosomes taken from either B cells or the urine that were the predominant source of the 3′ ends of uridylated endogenous miRNAs. This demonstrates that the 3′ ends of the miRNA sequence may be connected to a crucial sorting signal. The cytoplasmic lipid bilayers of the MVB limiting membrane are where the miRNAs with the greatest affinity to the raft-like area are accumulated. RNA-binding proteins transport miRNAs to be bound to this region. The specific binding motifs, like the GGAG pattern, may very well determine this transport. A spontaneous process of inward budding from the raft-like area takes place once the miRNA has attached to it, thereby producing ILVs and subsequently exosomes. The cytoplasmic leaflet of the membrane’s ceramide molecules, as well as the lysophospholipid and glycosphingolipid molecules of the luminal leaflet, may be necessary for the budding process (22) and hence the ExomiRs are released into the maternal circulatory system.

### Biodelivery of exomiRs

Fusion of the hydrophobic cytoplasmic leaflets of the exosome and plasma membrane is likely mediated by families of Soluble N-ethylmaleimide-sensitive factor Activating Protein Receptor (SNARE) and Rab proteins to produce a hemifusion stalk, thereby initiating the fusion of exosomes (23, 24). The exosome surface contains integrins, adhesion molecules, and lipid raft-like structures that facilitate contact, attachment, and fusion of the membrane with the target cell (25, 26). The formation of clathrin-coated vesicles during clathrin-mediated endocytosis, which is characterized by the participation of the triskelion scaffold (clathrin), occurs as a result of the sequential assembly of multiple transmembrane receptors and ligands (27). Most cell types exhibit this mechanism of the exosomal entrance, this involves internalized vesicles that uncoil and fuses with endosomes with Dynamin 2 forming the neck-like structure during invagination required for scission. Internalized vesicles then become uncoated and join endosomes. Clathrin-mediated endocytosis is one of the most conventional exosome uptake pathways. Thus the ExomiRs are transferred to the recipient cell. The cargo and exosome composition can also affect this tightly controlled process (27, 28).

One important endocytic method to move exosomes into the early endosome and affect their uptake is lipid raft-associated membrane invagination (29). By immobilizing exosomes on the cell surface at particular adherent locations, annexin AnxA2 increases lipid raft-mediated endocytosis (30), and flotillin, a component of lipid rafts, also favorably controls this process by associating with membrane micro domains enriched in cholesterol and sphingolipids. According to reports, the assembly of flotillin-1 and flotillin-2 causes membranes to experience curvature stress and creates caveola-like invaginations at the plasma membrane (28, 31).

## ExomiRs as placental function marker

**Figure 2:**
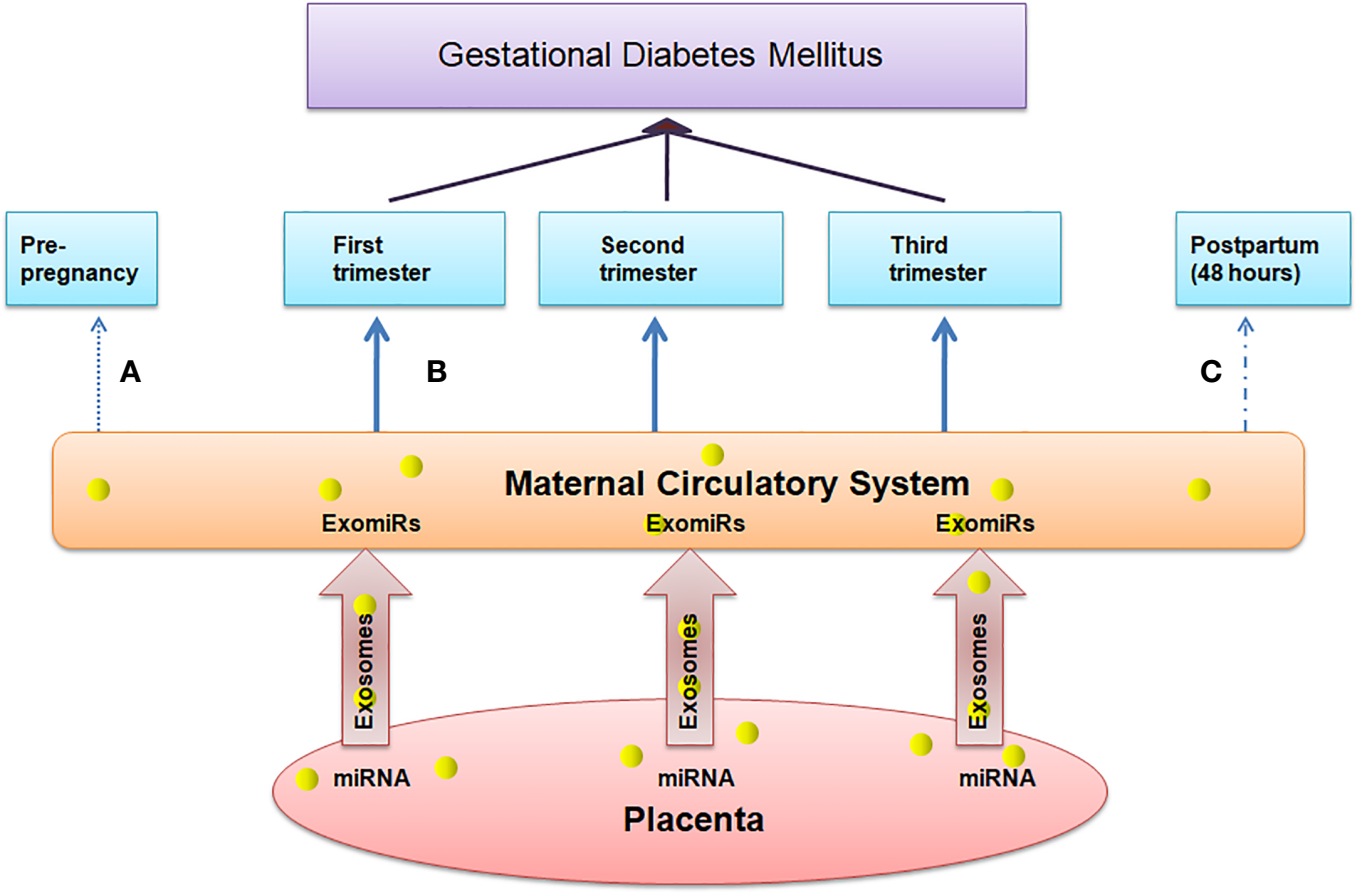
Depicts the association between the concentration of ExomiRs in maternal peripheral blood, which serves as a marker for placental health. **(A)** The concentration of ExomiRs in the maternal circulatory system is normal before pregnancy. **(B)** During complicated pregnancies like in GDM patients, the ExomiR concentrations, beginning from the first trimester, are observed to be altered, with most being upregulated and few being downregulated. **(C)** The ExomiR concentration is observed to be decreased after delivery and gets back to normal 48 hours postpartum.

Complications in pregnancy are associated with significant difference in the level of circulating exosomes and hence the concentration of ExomiRs in maternal plasma, their composition, and bioactivity from that of normal pregnancies (32). These exosomes that are released into maternal bloodare responsible for placental development and maternal immune tolerance (33). The human placenta is a transient organ that provides the required oxygen and food to the fetus and removes the waste products from the fetal blood by the umbilical cord thus the proper development and functioning of the placenta is required for normal deliveries making it an essential part of the maternal-fetal communication system. Angiogenesis under hypoxic conditions perhaps is one of the keystone signaling pathways, responsible for the zygote to undergo the process of blastulation and gastrulation, thereby promoting the fetal growth through tissue differentiation which is mediated by upregulated expressions of hypoxia-induced vascular-endothelial growth factor-mediated downstream signaling pathways involving but not limited to the expression of MMPs and their downstream signaling intermediates (34).Various evidences support the hypothesis of the role of ExomiRsin the origin of pregnancy-related complications in the early stages of gestation. The total concentration of ExomiRs helps us to indicate the difference between normal and pregnant women. Additionally, the concentration of these ExomiRsis altered in women with pregnancy-associated complications.ExomiRscanmodulate the gene expression by post-transcriptional repression or messenger RNA degradation in a sequence-specific manner (35) leading to the onset of various complicated pregnancy outcomes in pregnant mothers. The upregulation and downregulation of various ExomiRs make them efficient biomarkers, helping in the prognosis of various pregnancy complications with most of the ExomiRs being upregulated during complicated pregnancies and only some being downregulated acting as non-invasive biomarker due to several epigenetic modifications indicating placental health. A maternal-fetal communication system based on ExomiRs may exist as evidenced by the rapid alteration in maternal blood ExomiR levels within 48 hours following delivery (**Figure 2**). According to a study, placental and maternal ExomiRs can both move to the maternal circulation with compartment-specific expression from the placenta and even into the fetal compartment (36).

The studies that are currently provided, however,suggest that exosome biology is altered during pregnancy-associated complications. To determine the precise function of exosomes in complicated pregnancies, it is necessary to apply particular and well-characterized isolation approaches. Exosomal secretion by trophoblastic cells in the placenta to the maternal peripheral circulation is thought to be responsible for the higher rates of delivery of these vesicles during gestation, which also happens in response to various pathological conditions. Exosomes that have been isolated from the maternal circulation during a typical pregnancy also show variations in their bioactivity as the gestational age increases. A major cause for the variation in the bioactivity of miRs in different trimesters of pregnancy is due to environmental factors like hypoxia, obesity, signaling pathways as well as epigenetic modifications. Compared to exosomes obtained from the second and third trimesters of pregnancy, those from the first trimester are shown to be more bioactive in stimulating endothelial cell migration (32). This phenomenon could be crucial in identifying the abnormal placentation in complex pregnancies since it may be linked to the cellular origin and/or exosomel composition and thus act as potential prognostic biomarker for adverse perinatal outcomes (33). Exosomal protein composition has also been seen to be alter in Preeclampsia (PE) affected women (32).

## ExomiRs as an indicator of placental health in gestational diabetes mellitus

Several ExomiRs have been studied to interfere with the functioning of several genes and thus leading to insulin resistance in patients. This has been observed to be associated with several complications in patients like type 2 diabetes mellitus (T2DM) and Gestational Diabetes Mellitus (GDM) in pregnant women. ExomiRs also act as biomarkers that help in the early diagnosis of insulin resistance-related complications. Although the association between ExomiRs and GDM is yet to be uncovered, several genes are seen to be upregulated or downregulated making them efficient biomarkers for the prognosis of the disease. In a recent study, it was seen that screening patients for GDM in the second and the early third trimester helps us to indicate the specific pathophysiological placental features (7).

**Figure 3:**
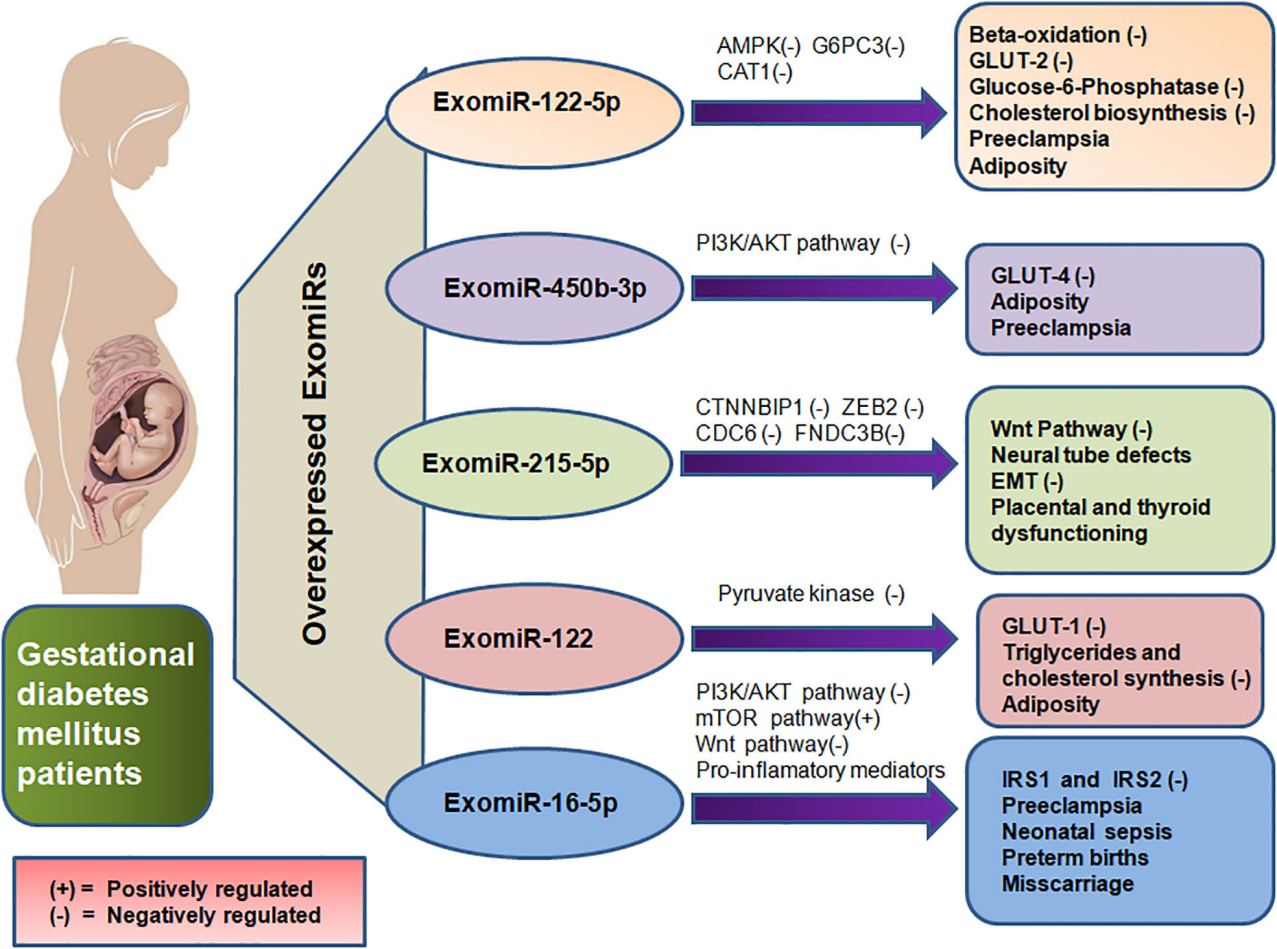
Depicts the overexpression of ExomiR in GDM patients, which results in their interference with the regulation of various genes and signaling pathways leading to several maternal and teratogenic outcomes thereby also acting as prognostic biomarkers for the GDM and its associated complications.

The upregulation of Exomir-122-5p in pregnant mothers with GDM shows their regulatory role in insulin resistance (37, 38), obesity (37) and regulation of glucose level (39, 40). This ExomiRis expected to prevent insulin from binding to the insulin receptor protein. GLUT-2 is anticipated to be inhibited by the ExomiR-122-5p which would result in reduced insulin production from pancreatic islet cells (41). Inhibition of the GLUT-2 receptor not only impairs glucose uptake by the cell but also causes the expression of other glucose transporters to be dysregulated resulting in dysglycemia during the course of pregnancy (42). The aggravated levels of ExomiR-122-5p found in GDM are expected to inhibit Adenosine 5′-monophosphate (AMP)-activated protein kinase (AMPK), which will thus prevent beta-oxidation and glucose transport (41). With its ability to activate insulin-sensitizing effects, AMPK is a phylogenetically conserved serine/threonine energy sensing kinase and is therefore a prime candidate for diabetes treatment. In addition to reducing hepatic glucose synthesis, it sends signals to enhance skeletal muscle glucose uptake and adipose (and other) tissue fatty acid oxidation (43). This inhibits the AMPK pathway and would also lead to hindering glucose uptake by the body cells thereby resulting in GDM. Moreover, the downregulation of beta-oxidation may also lead to adiposity in pregnant women (**Figure 3**). Exomir-122-5p also targets genes like Glucose-6-Phosphate Catalytic Subunit 3 (G6PC3) and Farnesyl-diphosphate farnesyltransferase 1 (FDFT1) essential for hydrolysis of glucose 6-phosphate in glycolysis and cholesterol biosynthesis respectively, impairing their proper functioning and leading to insulin resistance (44) and hence, GDM in patients.

ExomiR-16-5p regulates the PI3K/Akt, Wnt and mTOR signalling pathways, since these signalling pathways serve a key role in GDM (45–47). Upregulation ofExomiR-16-5p in GDM patients during the second trimester, IRS1 and IRS2 are negatively regulated, thereby showing the effect of ExomiR-16-5p on these genes. This impairs Wnt signalling pathway and may ultimately result in GDM by blocking the autophagic degradation of Dishevelled 2 (45, 48, 49) which modulates a glycogen synthase kinase allowing nuclear translocation of beta-catenin and subsequent activation of Wnt-target gene. The placental mammalian target of rapamycin (mTOR) signal is activated by ExomiR-16-5p overexpression, which encourages mitochondrial function, protein synthesis and the transport of nutrients like amino acids, improving fetal nutrition utilisation. Exosomes from GDM patients are more enriched in proteins targeting the mTOR signalling pathway than exosomes from those with normal glucose tolerance (**Figure 3**). Exosomes from GDM patients may therefore control placental nutritional capacity by stimulating the mTOR signal in the placental environment (50). It has been proposed that altered signalling of protein kinase B/Akt (Akt) and mTOR in human placental endothelial cells may be the cause of insulin resistance in pregnant women with GDM and their neonates (45, 51).

ExomiR-215-5pdownregulates the Catenin Beta Interacting Protein 1 (CTNNBIP1) gene which encodes the CTNNBIP1 protein that is a negative regulator of the Wnt signaling pathway leading to GDM (52). It is also an important factor in the mechanism of ADSCs-Exo-mediated protection against podocyte injury by suppressing ZEB2 transcription leading to Diabetic Nephropathy (DN) (53). DN is considered a cause of chronic hyperglycemic conditions as a result of GDM leading to the damaging of multiple organs including kidneys (54). As ExomiR-251-5p acts as a downstream regulator of ZEB2 (**Figure 3**), it increases the proangiogenic effect leading to the ExomiR causing Neural Tube Defects (NTD). NTD-associated genes like ZEB2, with a two-fold or greater change in expression control diabetes exposure to theembryos (55).Thus ExomiR-215-5p not only serves a key role in GDM but also in neonatal diabetes.

PI3K/AKT signaling pathway is inhibited by ExomiR-450b-3p, which maintains insulin-induced protein Forkhead Box protein O1 (FOXO1) rejection and thereby impairing the GLUT-4 trafficking leading to impaired glucose tolerance (56). Additionally, the Akt signaling pathway phosphorylates FOXO1 transcription factors promoting adipogenesis as FOXO1 prevents the maturation and differentiation of adipocytes thereby playing a significant role in obesity (57).This leads to an increase in the Basal Metabolic Index (BMI) of the pregnant mother and hence increases the risk stratification of obesity-related pregnancy outcomes like GDM (**Figure 3**). Hyperlipidemia in the first trimester of pregnancy might lead to the development of GDM in the second trimester (58).

ExomiR-122 controls the expression of the GLUT-1 receptor by downregulating pyruvate kinase, thereby hampering the glycolytic fluxes and subsequently decreasing glucose metabolism (54). The major transporter for glucose transfer in the placenta, GLUT-1, is essentially expressed in the endothelial cells of the placental villi and syncytiotrophoblast (59). Syncytiotrophoblasts are primarily responsible for nutrient and gas exchange in the placenta. The downregulation of GLUT-1 by ExomiR-122 leads to a decrease in GLUT-1-mediated glucose transport activity (**Figure 3**) thereby leading to GDM (60).

## Preeclampsia-associated exomiRs

GDM is most commonly linked to its pathophysiological outcome, Preeclampsia (PE),as a result of oxidative stress, pro-inflammatory factor release, and vascular-endothelial dysfunction. The occurrence of the hypertensive disorder, ie.,PE is positively correlated with blood glucose levels. The association between GDM and PE is not specific to obesity or primigravida but the association between the two increases with obesity and specifically gestational weight gain (61). In the early stages of pregnancy, new blood vessels develop for the supply of oxygen and nutrients to the fetus. These blood vessels do not work or develop properly in women with PE and this, in turn, leads to dysregulation of blood pressure in women with PE, which is generally determined in the second trimester of pregnancy.

ExomiR-122-5p has been observed to have a crucial role in metabolism of cholesterol by targeting Cationic amino acid transporter 1 (CAT1), which transports cationic amino acids (**Figure 3**) and can be linked to dyslipidemia in PE (62). ExomiR-122-5p elevated levels may be attributed to the abnormal glycosylation of the mucin-type O-glycosylated antigen, which is interconnected with the augmented maternal inflammatory responses seen in severe PE (63). Furthermore, recent research has linked the abnormal glycosylation of proteins in PE to the synthesis of new proteins that are involved in hepatic and renal dysfunction, implying that placenta-derived exosomes may be engaged in the end-organ abnormalities associated with severe forms of PE (64).

PI3K/Akt pathway overexpression by ExomiR-16-5p is involved in the osteogenic differentiation of cells thereby inhibiting the pro-apoptotic protein Cyt C, Apaf-1 and Bax. Nephronectin, an osteogenesis enhancer, is silenced which suppresses the early phases of osteoblast development in pregnant women. However, the bindingExomiR-16-5p to the 3’-UTR of nephronectin releases GalNT-7, which is another target also known to glycosylate proteins, including nephronectin to become active (65, 66). This increases the risk of PE (**Figure 3**)  (67).

Overexpression of ExomiR-215-5p in pregnant women inhibits the proliferation and migration of trophoblasts during PE by limiting CDC6 (**Figure 3**). CDC6 gene codes for a protein, CDC6 that is required for the process of DNA replication. An examination of the cell cycle distribution of trophoblast cells reveals that the number of cells in the G1 phase visibly increases whereas the number of cells in the S-phase decreases significantly (68).ExomiR-215-5p also prevents the epithelial-mesenchymal transition (EMT) by impairing CDC6 *via* the epigenetic downregulation of E-cadherin expression (69).

Hyperlipidemia caused as a result of inhibition of PI3K/AKT signaling by ExomiR-450b-3p as a result of phosphorylation of FOXO1 transcription factor (57, 58) not only leads to adiposity-induced insulin resistance by impairing with GLUT-4 trafficking (56), but also the hypertensive disorder of PE as a result of obesity(**Figure 3**). Similarly, ExomiR-122-induced inhibition of GLUT-1 decreases glucose metabolism by downregulating pyruvate kinase (70) thereby also impairing the synthesis of triglycerides and cholesterol leading to obesity (71) and hence the onset of PE (60).

In PE, early insufficient trophoblast invasion causes improper spiral artery remodeling leading to placental ischemia and oxidative stress causing morbidity and mortality in mothers and infants and is considered a pregnancy-specific seizure disorder which is accompanied by the onset of proteinuria, and elevated blood pressure serving as recognition factors (72). Severe PE may lead the patient to undergo Cesarean delivery (C-section) (73).

## ExomiRs-associated with other maternal and teratogenic outcomes

Several ExomiRs have been seen to have an association with adverse pregnancy outcomes with some of the placental origins, some are pregnancy state-specific and others are involved in a pathophysiological state of diabetes, which is associated with other severe pregnancy-related outcomes. Disruption of the tightly regulated endocrine system through sustained perturbation of hypothalamus-pituitary signaling cascade may lead to maternal complications including but not limited to GDM, preeclampsia, and hypothyroidism, which could either result in miscarriages, preterm births, pregnancy complications as well as increased pre-disposition of the offspring to neonatal sepsis (74).

During early adipogenesis, ExomiR-215-5p serves as a repressor of adipocyte differentiation *via* post-transcriptional regulation of Fibronectin type III Domain Containing 3B(FNDC3B) (52), which serves a ubiquitous role in the placenta. Decreased levels of ExomiR-215-5plead to ectopic pregnancy in the early stages of pregnancy accompanied by abdominal pain or vaginal bleeding (75). FNDC3B also serves a ubiquitous role in the thyroid leading to the onset of hypothyroidism. A study also shows that circulating ExomiR-215-5p in women in the second trimester of pregnancy was determined to be associated with the birth weight-at-gestational stage (76). Downregulation of NTD-associated genes like ZEB2 is caused as a result of ExomiR-251-5p upregulation (**Figure 3**), thereby regulating neural tube development hence altering embryonic expression leading to NTDs. These NTDs, in some cases, may progress from a wavy neural tube to spina bifida in various locations of the neural tube leading to exencephaly and craniorachischisis. This can be detected by the upregulation of ExomiRs in the mother’s blood (55). ExomiR-16-5p enhances the secretion of proinflammatory cytokines in the human placenta by inhibiting the Apelin signaling pathway, where Apelin serves as a potent inhibitor of proinflammatory mediators thereby activating pro-labor hormones and cytokines including IL-1, IL-6, IL-8, and TNF-α. This leads to preterm births and C-sections (77). An elevation in the levels of proinflammatory cytokines likeIL-6 and IL-8 in the placenta also acts as a precursor for an increased risk of neonatal sepsis as a result of autophagy in the placenta (78). This can be well determined by the leukocyte count of the pregnant mother (79). Additionally, premature infants are more prone to Bronchopulmonary Dysplasia as a result of sepsis (80). Moreover, obesity induced by the overexpression of ExomiR-450b-5p and ExomiR-122 may also lead to cases of miscarriage among pregnant women (81).

## Can epigenetic markers be prospected as theranostic target?

Not only ExomiRs aid to act as a potential causative agent for insulin resistance during pregnancy but there are certain ExomiRs that when overexpressed, lead to overcoming insulin resistance in patients by influencing glucose uptake.

Overexpression of ExomirR-221 targetsp21-activated kinase (PAK1) which regulates the proliferation and suppresses apoptosis of beta cells of islets of the pancreas thereby regulating insulin secretion (63, 82). ExomiR-96 when overexpressed in cells was found inversely correlated with the rise in blood glucose level in GDM conditions. It is also found to target PAK1 specifically like ExomiR-221 (82, 83). Therefore, these can possibly act as an effective tool to resist GDM-induced insulin resistance. These ExomiRs influence the cells’ enhanced insulin selection leading to insulin secretion and also enhances the proliferative activity of cells. Their effect on cells’ viability and apoptosis were partially reversed by PAK1 which meant that PAK1 was necessary for its protective impact on islet beta cells (65). Fasting hyperglycemia and severe glucose intolerance were also found to be present in PAK1-deficient individuals (83).

It was observed in Human Primary Trophoblasts (PHT) that overexpression of ExomiR-515-5p eventually significantly stimulates glucose uptake by cells. It regulates the functioning of Insulin like Growth Factor 1 Receptor (IGF1R) thereby stimulating glucose uptake (84). Proteins linked to glycolysis were differentially expressed in ExomiR-515-5p overexpressed PHT cells, according to a proteomics investigation (85). These data imply that in GDM patients, increased placental nutrition transfer may be a result of adipose tissue ExomiR515-5p mediated placental glucose uptake (85, 86).

Thus, these ExomiRs can not only be used as potential non-invasive biomarkers for the prognosis of GDM but also their ability to increase in glucose uptake makes them significant to be used as a clinical tool for reducing the risk of GDM and related pregnancy complications (87, 88).

## Conclusion

The rise in the incidence of GDM, in turn gives rise to increasing number of maternal and fetal complexities with adverse consequences. To manage the burden of GDM, clinical interventions supplemented with bio-behavioral health-based interventions could significantly alleviate the clinical prognosis of GDM in the world. There have been numerous studies that characterized the expression of circulating miRNAs or ExomiRs from pregnant women, thus suggesting their role in pathogenesis of GDM, however, their potential molecular mechanisms are still unknown.

The main purpose of this review is to assess a panel of ExomiRs being prospected for early diagnosis of GDM in communities with multiple ethnicities, socio-cultural norms and lifestyle choices. This is very pertinent in Indian subcontinent where there are five distinct centers of origin having population with varying socio-cultural norms and lifestyle habits, which could perhaps create a variation in the existing panel of exomiRs which is being prospected. The identification of the race- and niche-specific ExomiRs as biomarkers can help in predicting and diagnosing GDM in the first trimester of pregnancy to avoid any pregnancy-associated complications through timely intervention at the community level.

The aggregation of hyperglycemic signals from the pregnant women at the community level to detect recurrent and emergent hotspots of GDM poses a major challenge for healthcare professionals. To this end, the deployment of a federated learning-based system to detect GDM using m-health platforms will not only facilitate optimal detection of GDM but also provide insights into the automated allocation of clinical resources, along with identification of the impending risk factors (precursors) contributing to the GDM epidemic, even in remote locations.

**Figure 4:**
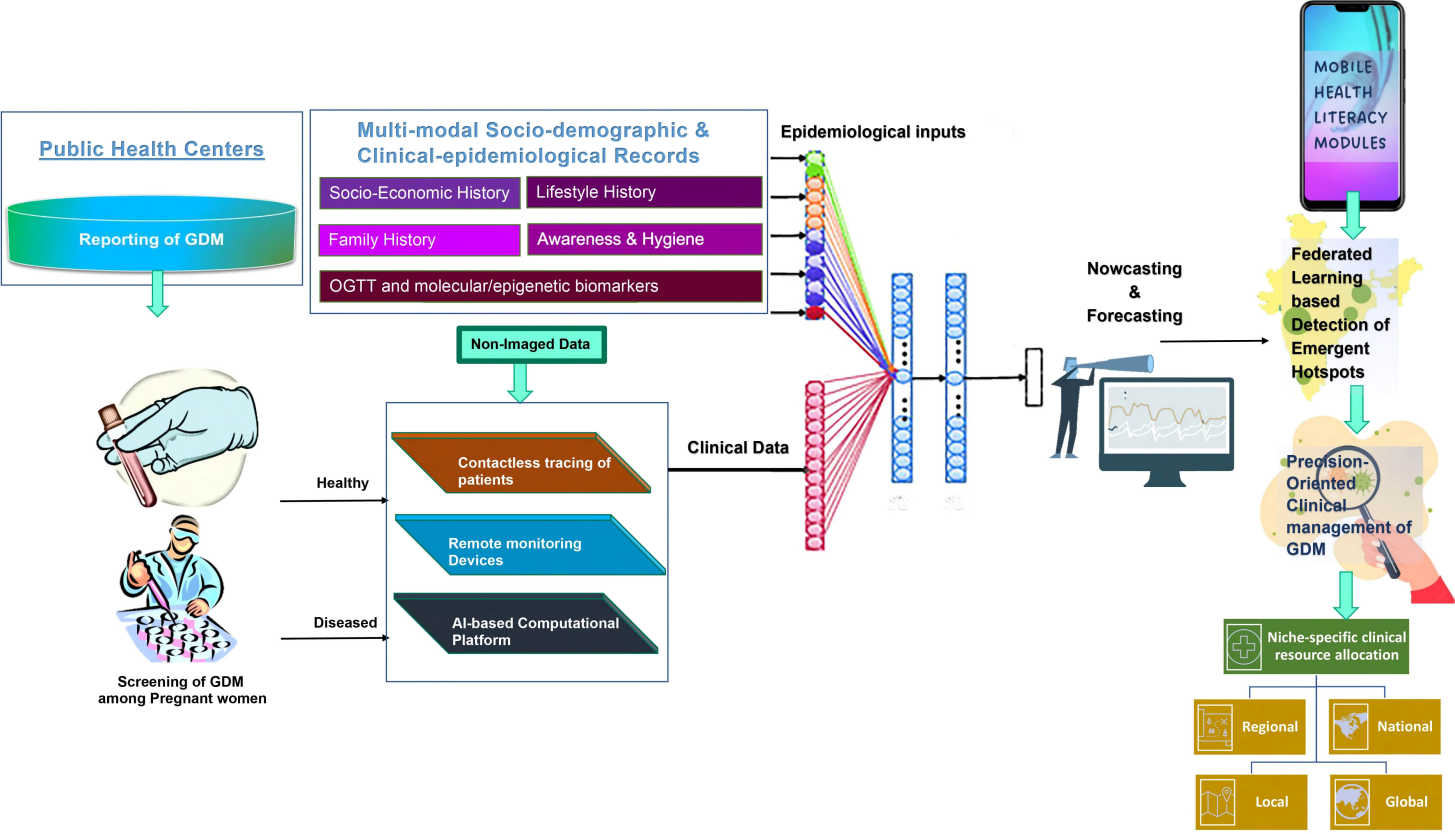
Shows the integration of digitized signals from the molecular markers, epidemiological data, as well as clinical data for the development of AI-enabled nowcasting and forecasting system, which when combined with the mobile-health-based health literacy modules, will help in the deployment of the federated learning-based detection of emergent hotspots of the Gestational diabetes (GDM)and its associated complications among pregnant women for the precision-oriented clinical management of the disease. This AI-enabled platform also forms the rationale for niche-specific allocation of the clinical resources at the community level.

We believe that the use of AI-enabled dashboards (**Figure 4**) endowed with digital signals of hyperglycemia, as well as epigenetic/molecular biomarkers, will facilitate precision-oriented large-scale screening of GDM in both rural, semi-urban, and urban milieus. This will facilitate the remote connection of the physicians’ team with the patients along with the provision of health literacy modules to the vulnerable population. The heuristic capabilities of this iterative and interactive dashboard will be proactively used to develop nowcasting and forecasting strategies towards the development of niche-specific data-driven surveillance system, integrating all the stakeholders of the healthcare ecosystem for developing community empowering bottoms-up policies/programs to help the communities at local, regional, national, and global levels.

## Author contributions

TM, RG, SK, ST, PR, and RJ designed the concept of the manuscript. TM and RG wrote the initial draft of the ms and made the concept figures with help from AU, PR, and RJ. SK, ST, PR, and RJ reviewed the paper extensively and provided their critical inputs in refining the concepts of the ms. RJ, TM, and RG were responsible for the overall concept and quality of the ms. All authors contributed to the article and approved the submitted version.
